# Comparison of haematology and biochemistry parameters in healthy South African infants with laboratory reference intervals

**DOI:** 10.1111/tmi.13009

**Published:** 2017-12-04

**Authors:** B.‐M. Schmidt, M. Tameris, H. Geldenhuys, A. Luabeya, E. Bunyasi, T. Hawkridge, J. B. McClain, H. Mahomed, T. J. Scriba, H. McShane, M. Hatherill

**Affiliations:** ^1^ South African Tuberculosis Vaccine Initiative Institute of Infectious Disease and Molecular Medicine and Division of Immunology Department of Pathology University of Cape Town Cape Town South Africa; ^2^ Department of Health South Africa; ^3^ Aeras Rockville MD USA; ^4^ Nuffield Department of Clinical Medicine Jenner Institute University of Oxford Oxford UK

**Keywords:** reference interval, haematology, biochemistry, South African infants, clinical trial, intervalle de référence, hématologie, biochimie, nourrissons sud‐africains, essai clinique

## Abstract

**Objective:**

Paediatric laboratory reference intervals used in Africa and Asia may be derived from historical intervals of predominantly Caucasian infants in Europe or North America. These intervals may therefore not be compatible with the range of normality for developing country populations. We aimed to compare haematology and biochemistry parameters in healthy South African infants with local laboratory reference intervals.

**Methods:**

We compared the baseline haematology and biochemistry results of 634 (316 male and 318 female) HIV‐unexposed infants, aged 3–6 months, living in a rural area of the Western Cape Province, South Africa, against laboratory reference intervals supplied by the South African National Health Laboratory Services (NHLS). We calculated the percentage of observed values out of bound (in terms of lower and upper limits) compared to laboratory reference intervals.

**Results:**

Of the 634 healthy infants screened, 316 (49.84%) were male and 318 (50.16%) female. A majority (91.05%) had platelet counts above the laboratory reference interval upper limit (350 × 10^9^cells/l), while over half, 54.85% and 56.98% had mean corpuscular volume (MCV) and mean corpuscular haemoglobin (MCH) values below the lower limits of 77.0–105.0 fl and 26.0–34.0 pg, respectively. A small proportion were outside the reference limits for haematocrit, namely 15.71% below and 7.14% above the normal limits of 0.31–0.38 l/l. For male and female infants, 33.65% and 18.04% of alkaline phosphatase (ALP) values and 7.01% and 14.56% of alanine transaminase (ALT) values were above the upper limits, respectively. For male infants, 10.83% of gamma‐glutamyl transferase (GGT) values, and for female infants, 31.11% of GGT values were below the lower limits of 12 U/l for males and 15 U/l for females. We observed no significant deviations (>10% out of bound) from NHLS reference intervals in the remaining haematology and biochemistry parameters measured.

**Conclusions:**

Haematology and biochemistry parameters in apparently healthy South African infants deviate frequently from national laboratory reference intervals, including abnormalities consistent with subclinical hypochromic microcytic anaemia. It is important that clinical laboratory reference intervals for children are derived locally, rather than being adopted from Caucasian norms in developed countries, because clinical trials of vaccines, drugs and diagnostics are increasingly conducted in sub‐Saharan Africa.

## Abstract (fr)

**Objectif:**

Les intervalles de référence des laboratoires pédiatriques utilisés en Afrique et en Asie seraient dérivés d'intervalles historiques de nourrissons à prédominance caucasienne en Europe ou en Amérique du Nord. Ces intervalles pourraient donc ne pas être compatibles avec l’étendue de la normalité pour les populations des pays en développement. Nous avons cherché à comparer les paramètres hématologiques et biochimiques chez des nourrissons sains sud‐africains avec les intervalles de référence des laboratoires locaux.

**Méthodes:**

Nous avons comparé les résultats hématologiques et biochimiques de 634 nourrissons non exposés au VIH, âgés de 3 à 6 mois, vivant dans une zone rurale de la province du Wertern Cap en Afrique du Sud aux intervalles de référence fournis par les Services Nationaux de Laboratoire de Santé (NHLS) d'Afrique du Sud. Nous avons calculé le pourcentage des valeurs hors limites observées (en termes de limites inférieures et supérieures) par rapport aux intervalles de référence de laboratoire.

**Résultats:**

Sur les 634 nourrissons sains examinés, 316 (49,84%) étaient masculins et 318 (50,16%) féminins. Une majorité (91,05%) avait des numérations plaquettaires au‐dessus de la limite supérieure de l'intervalle de référence de laboratoire (350 x 10^9^ cellules/L), tandis que plus de la moitié, 54,85% et 56,98% avaient des valeurs moyennes de volume corpusculaire et d'hémoglobine corpusculaire en dessous des limites inférieures de 77.0‐105.0 fl et 26.0‐34.0 pg, respectivement. Une petite proportion était en dehors des limites de référence pour l'hématocrite, à savoir 15,71% en dessous et 7,14% au‐dessus des limites normales de 0,31 l/L ‐ 0,38 l/L. Chez les nourrissons masculins et féminins, 33,65% et 18,04% des valeurs de la phosphatase alcaline (ALP) et 7,01% et 14,56% des valeurs de l'alanine transaminase (ALT) étaient respectivement au‐dessus des limites supérieures. Chez les nourrissons masculins, 10,83% des valeurs du gamma glutamyl transférase (GGT) et chez les nourrissons féminins, 31,11% des GGT étaient en dessous des limites inférieures de 12 U/L pour les garçonnets et de 15 U/L pour les fillettes. Nous n'avons pas observé d’écart significatif (> 10% hors limite) par rapport aux intervalles de référence des NHLS pour les autres paramètres hématologiques et biochimiques mesurés.

**Conclusions:**

Les paramètres hématologiques et biochimiques des nourrissons sud‐africains sains s’écartent fréquemment des intervalles de référence nationaux de laboratoire, y compris des anomalies cohérentes avec l'anémie microcytaire hypochrome sub‐clinique. Il est important que les intervalles de référence des laboratoires cliniques pour les enfants soient dérivés localement plutôt qu'adoptés à partir des normes caucasiennes dans les pays développés, car les essais cliniques de vaccins, médicaments et diagnostics sont de plus en plus menés en Afrique subsaharienne

## Introduction

Age‐ and sex‐appropriate local reference intervals are important for the correct interpretation of laboratory tests needed for clinical management of patients and for screening and safety assessment of children participating in clinical research in developing countries 1, 2. However, paediatric laboratory reference intervals provided in Africa and Asia may be derived from historical reference intervals, reported from predominantly Caucasian infants in Europe or North America 3. Incorrect interpretation of normality or abnormality, based on these laboratory reference intervals, might lead to unnecessary investigation and treatment, or conversely, appropriate intervention may be withheld.

Previous studies have shown that laboratory reference values may differ from values observed in healthy African children 3, 4, 5. Two studies, one focusing on Malawian and Ugandan infants and another on Zimbabwean infants, have shown abnormalities in haemoglobin and neutrophil values of healthy, HIV‐unexposed African infants when laboratory values derived from European or American populations were applied 3, 4. Possible reasons for such deviations from laboratory reference intervals include ethnicity, gender, age, dietary patterns, genetics and environmental pathogens 6, 7. The public health impact of these deviations would depend on the frequency and severity of the abnormality in the local population. In the context of clinical studies, the application of reference intervals that are not well‐suited to the local population may result in healthy volunteers being excluded from participation in trials, which may lead to misrepresentations of the study community. Further, data generated to establish safety and clinical parameters in paediatric clinical trials of drugs, vaccines and diagnostics may not accurately reflect the true scope and severity of adverse events related to the investigational products and devices.

The frequency and severity of deviations from laboratory reference intervals have not been fully evaluated in a population of apparently healthy South African infants 8. Haematology reference intervals were previously established for the Black population of the Witwatersrand in 1987; however, these were never implemented by the National Health Laboratory Services (NHLS) (previously the South African Institute for Medical Research), because of constraints in the laboratory information system at the time. Currently, the South African NHLS uses reference intervals by Mendelow *et al*. (unpublished data, 1985), derived from Caucasian subjects 9. The primary objective of this study was to compare haematology and biochemistry parameters in apparently healthy South African infants with national laboratory reference intervals provided by the NHLS, to inform guidelines for clinical care and to provide appropriate standards for clinical research in children in this developing country setting.

## Methods

The South African Tuberculosis Vaccine Initiative (SATVI), operating from a field site in the Western Cape Province of South Africa, has enrolled more than 5000 healthy infants, adolescents and adults into phase 1‐2b tuberculosis vaccine trials since 2005. Early phase trials have strict eligibility criteria including specified haematology and biochemistry parameters; adverse events are usually assessed according to tables from a developed country source, for example the Division of AIDS Table for Grading the Severity of Adult and Paediatric Adverse Events of December 2004, (http://rcc.tech-res.com/tox_tables.htm). This analysis was carried out using baseline screening data from two novel tuberculosis vaccine trials, conducted by SATVI between 2008 and 2012, in infants from the greater Worcester area of the Western Cape, South Africa. The trial protocols were approved by the University of Cape Town Faculty of Health Sciences Human Research Ethics Committee and registered with the South African National Clinical Trials Register (DOH‐27‐0109‐2654 and DOH ‐27‐0611‐3044) and ClinicalTrials.gov (NCT00953927 and NCT01198366); the findings have been reported previously 10, 11. Both trials underwent external monitoring and were subjected to Data and Safety Monitoring (DSMB) review, and trial data were collected and captured according to Good Clinical Practice (ICH GCP).

Healthy infants were recruited from the general population using vaccination clinic records, birth registers and referrals from community contacts and other participants. Informed consent was signed by the mother or legal guardian before any study procedures were performed. Mothers <18 years old signed assent and their legal guardian consent. Infants were required to be Bacille Calmette Guerin (BCG) vaccinated at birth, HIV‐unexposed, have neither a maternal history of tuberculosis disease or exposure to an infectious household contact, confirmed by either negative tuberculin skin test (TST) ≤10 mm or interferon gamma release assay (IGRA). General good health was determined by a study clinician on medical history and examination at the time of screening. Weight‐ and age‐specific *z*‐scores were computed from measurements taken at the visit. Only infants between the 3rd and 97th percentile for weight for age were included.

Study investigators were responsible for continuous medical care and assessment of infants and assessed all blood chemistry and haematology parameters using reference intervals supplied by the processing laboratory, NHLS, the sole provider of diagnostic pathology services for the public sector in South Africa. Haematology tests (full blood count and differential cell count) were performed with the Sysmex XN10 instrument (manufactured in Germany), biochemistry tests were performed with the Roche Cobas 6000 instrument (manufactured in Japan). Both instruments used German reagents. No changes were reported in these instruments during the period under study. Haematology and chemistry tests were performed according to ISO 15189 2007 requirements in terms of technical competency and quality management.

Our analysis focused on the haematology and biochemistry results of 634 apparently healthy infants (318 females and 316 males) aged between three and six months, who were enrolled into two clinical trials with similar inclusion and exclusion criteria. The health status of infants was determined through medical history, physical examination, HIV testing, full blood count and differential, liver and renal function parameters, and a negative QuantiFERON^®^–TB Gold In‐Tube test. All parameters analysed were obtained at screening (or baseline), prior to receipt of study investigational product.

Maternal HIV status was checked on the infant's Road to Health Card and infant HIV status confirmed by HIV PCR at the time of screening. Mothers were not screened but the infant's Road to Health Card was perused to exclude any potential congenital condition including syphilis.

The measured haematology parameters for full blood count were white blood cells (WBC), red blood cells (RBC), haemoglobin (HB), haematocrit, mean corpuscular haemoglobin (MCH), mean corpuscular volume (MCV), mean corpuscular haemoglobin concentration (MCHC) and platelets. Biochemistry measures for total bilirubin, alkaline phosphatase (ALP), alanine transaminase (ALT), aspartate transaminase (AST) and gamma‐glutamyl transferase (GGT) were also obtained.

### Statistical analytic methods

The normal distribution for baseline screening data collected from two SATVI trials were tested. Given a non‐normal distribution, median and interquartile ranges were calculated as well as the confidence intervals of the 2.5 and 97.5 percentiles of each variable. For each variable, we also calculated the percentage of observed values outside its lower and upper limits as compared to the NHLS laboratory reference intervals 12.

## Results

Of the 634 infants included in the analysis, 316 (49.9%) were male and 318 (50.2%) were female infants aged between three and six months. The majority (464, 73.2%) were Mixed Race, and 169 (26.7%) infants were Black; overall their median birth weight was 3.00 kg (2.66–3.31) with a birth weight‐for‐age *Z*‐score of 0.02 (−0.64 to 0.63) (weight was measured at screening and at each subsequent clinic visit).

Of the 13 haematology and biochemistry parameters measured, seven parameters had values below or above the NHLS reference intervals in 10% of infants (Table 1).

**Table 1:** Percentage (%) of observed values out of bounds compared to South African National Health Laboratory Services (NHLS) laboratory reference intervals, with calculated reference intervals (95% confidence interval) based on observed values

Haematology variables (units)	Median (IQR) (range)	NHLS intervals	% low	% high	Calculated 95% CI
White blood cell count (×10^9^/l)	10.92 (8.97, 13.50) (4.31–24.71)	5.50–18.00	0.95	4.29	6.11–19.08
Red blood cell count (×10^12^/l)	4.38 (4.09, 4.63) (1.42–5.73)	2.70–5.90	0.32	0.00	3.63–5.15
Haemoglobin (g/dl)	11.1 (10.5, 11.70) (8.4–13.5)	9.10–13.10	1.75	1.59	9.3–12.8
Haematocrit (l/l)	0.33 (0.31, 0.35) (0.17–0.87)	0.31–0.38	15.71	7.14	0.28–0.39
Mean corpuscular volume (fl)	76.4 (73.5, 79.4) (55.1–91.55)	77.00–105.0	54.85	0.00	66.8–85.3
Mean corpuscular haemoglobin (pg)	25.7 (24.4, 26.8) (18.5–74.1)	26.0–34.0	56.98	0.48	21.5–28.9
Mean corpuscular haemoglobin concentration (g/dl)	33.4 (32.4, 34.4) (21.9–37.5)	29.1–37.0	0.32	0.16	30.4–35.9
Platelet count (×10^9^/l)	522 (424, 625) (142–1003)	140–350	0.00	91.05	281–858

Biochemistry variables (units)	Median (IQR) (range)	NHLS intervals	% low	% high	Calculated 95% CI
Total bilirubin (μmol/l)	3 (3, 4) (1–12)	0–21	0.00	0.00	2–7
Alkaline phosphatase (U/l)	278 (235, 326) (22–959)	75–316 (male) 124–341 (female)	0.32 0.00	33.65 18.04	171–487 176–475
Gamma‐glutamyl transferase (U/l)	19 (14, 26) (6–119)	12–122 (male) 15–132 (female)	10.83 31.11	0.00 0.00	9–58 8–70
Alanine transaminase (U/l)	21 (17, 26) (6–83)	4–35 (male) 3–30 (female)	0.00 0.00	7.01 14.56	11–49 11–52
Aspartate transaminase (U/l)	40 (35, 46) (11.9–87)	0–65 (male) 0–79 (female)	0.00 0.00	7.36 4.92	28–63 29–68

### Haematological parameters

**Figure 1:**
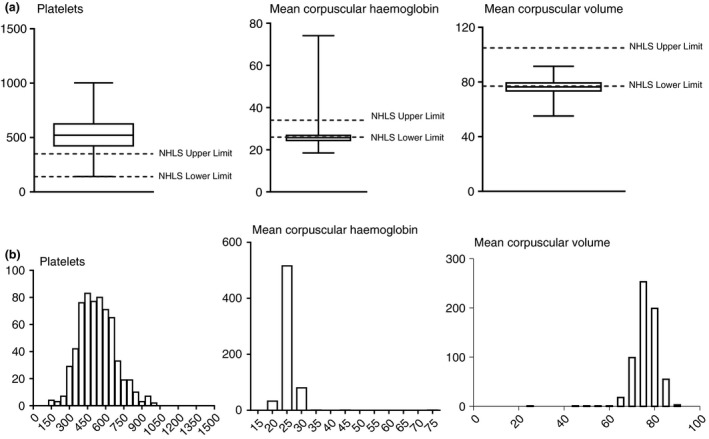
(a) Box‐and‐whisker plots showing variation between infant platelet count (×10^9^/l), mean corpuscular haemoglobin (pg) and mean corpuscular volume (fl), compared to South African National Health Laboratory Services (NHLS) laboratory reference intervals. (b) Distribution plots of observed infant platelet count (×10^9^/l), mean corpuscular haemoglobin (pg) and mean corpuscular volume (fl) values.

Most infants (*n* = 574) (91.05%) had platelet counts above the laboratory upper limit of normal (ULN) (350 × 10^9^ cells/l); over half (*n* = 360; 56.98%) had MCH values below the lower limit of normal (LLN) (26.0–34.0 pg); and 345 (54.85%) had MCV values below the LLN (77.0–105.0 fl). Amongst those with elevated platelet values, 310 (54.01%) had decreased MCV values and 316 (55.05%) decreased MCH values. In total, 417 (66.19%) children showed a combination of high platelet values and low MCV and MCH values. Regardless of platelet values, 506 (80.06%) had a combination of low MCV and low MCH values. Amongst those infants that had platlet values above the ULN, 281 (88.92%) were male and 292 (91.82%) were females, and 152 (89.94.0%) Black African infants and 422 (90.95%) Mixed Race infants. These differences were not significant (Figure 1.)

Significantly, more males had MCV values below the LLN, 199 (62.78%) males *vs*. 146 (46.06%) (*P* = 0.001). Similarly, for MCH values, 202 (64.13%) males and 156 (49.21%) females (*P* = 0.0001) had values below the lower limit. There was no significant difference for race for either MCV or MCH values. We also observed that 15.71% of all infants had haematocrit values below the LLN and and 7.15% above the ULN of 0.31 and 0.38 l/l, respectively. For the remaining haematological parameters, that is WBC, RBC, HB and MCHC, there were no clinically significant deviations from NHLS reference intervals.

### Biochemical parameters

**Figure 2:**
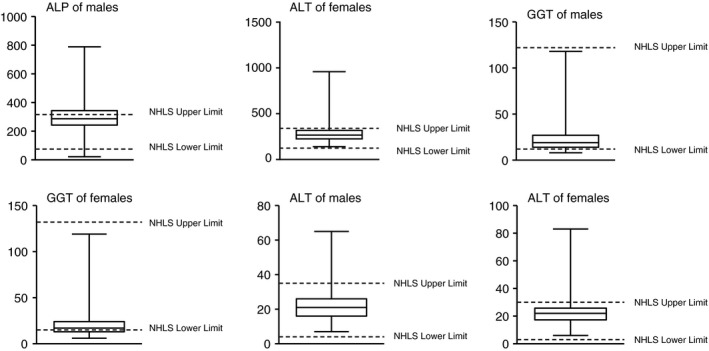
Box‐and‐whisker plots showing variation between infant alkaline phosphatase (U/l), gamma‐glutamyl transferase (U/l) and alanine transaminase (U/l) values by gender and South African National Health Laboratory Services (NHLS) laboratory reference intervals.

Infant values for ALP, GGT and ALT were commonly identified as abnormally low or high, compared to the laboratory reference intervals with 106 males and 57 females, having ALP measurements above the gender‐specific laboratory limits of 75–316 U/l and 124–341 U/l, respectively; males (33.54%) contributed a significantly higher proportion than females (17.92%) (*P* < 0.00). There was a significant difference in the proportions of GGT measurements below the lower limits (12 U/l for males and 15 U/l for females) contributed by males (10.83%) *vs*. females (31.11%) (*P* < 0.00). The proportions of ALT measurements above the upper limits (35 U/l for males and 30 U/l for females) differed significantly between males (7.01%) and females (14.56%) (*P* = 0.002). See Figure 2 **.** When female and male results for ALP, GGT and ALT were stratified by race, there were no significant differences.

We observed no clinically significant deviations (>10%) from NHLS reference intervals for total bilirubin (0.00%) and AST (for males 7.36% and for females 4.92% above the ULN).

## Discussion

The majority of clinically healthy, HIV‐unexposed infants in this rural region of the Western Cape Province of South Africa demonstrated apparent haematological and biochemical abnormalities, if the national age‐specific laboratory reference intervals are used as the standards for normality.

Specifically, more than 90% of infants had elevated platelet counts and more than half had decreased MCV and MCH values. Elevated platelet counts in infancy may be due to secondary thrombocytosis with no evidence of a coagulation disorder 13, 14. A common cause of secondary thrombocytosis and microcytosis in children is iron deficiency anaemia 15. Given the evidence of associated hypochromic microcytosis, and in the absence of definitive biochemical tests of iron storage, we hypothesise that many of these apparently healthy infants suffer from sub‐clinical, early iron deficiency anaemia. Iron deficiency anaemia in the developing world may be due to insufficient dietary intake, malabsorption of iron, or iron loss from subclinical gastrointestinal bleeding frequently caused by helminths 16. It is conceivable that this study population is genetically more predisposed to secondary thrombocytosis and hypochromic microcytosis, compared to the population from which the NHLS laboratory references intervals were originally derived.

A significant proportion of ALP and ALT values were higher than laboratory upper limits, while GGT values were unusually low in one‐third of females. These findings, in otherwise healthy infants without clinical evidence of hepatic disease, are consistent with the literature suggesting that laboratory reference intervals derived from European or American populations may not be applicable to Africans 17. Minor hepatic enzyme abnormalities, compared to these laboratory reference intervals, should therefore be interpreted with caution in South African infants.

A strength of this study is that the samples used for the analysis were obtained from two different clinical trials, which recruited healthy infants using clinic records, direct approach and community contacts over a period of four years. The results are felt to be representative of the infant population of the Western Cape Province, South Africa. A limitation of the study was that only protocol‐defined haematology and biochemistry parameters were measured. Additional measurements of iron storage, such as serum ferritin, were not available to confirm the hypothesis of sub‐clinical iron deficiency. Data on infant feeding practices were not captured.

It is apparent from our data that evaluation of haematological and biochemical parameters from cohorts of clinically healthy infants may demonstrate previously unsuspected trends in health and disease at a population level, as is the case with apparently widespread thrombocytosis and hypochromic microcytosis in this study population. These data might be used to inform healthcare providers and guide public health interventions in affected communities, including anaemia screening and iron and multivitamin supplementation. Conversely, data from healthy infants may suggest that deviations from existing reference standards are not appropriate triggers for medical intervention, which, in the absence of a reasonable hypothesis for widespread subclinical liver dysfunction, appears to be the case for the observed abnormalities in hepatic enzymes in this study. These findings also have significance for future clinical research in children living in developing countries of Africa, where it is apparent that historical laboratory reference intervals derived from Caucasian populations may not be appropriate standards to inform the safety database for investigational products. Clinical trials of vaccines, drugs and diagnostics are increasingly conducted in sub‐Saharan Africa, and therefore, it is important that clinical laboratory reference intervals for children are derived locally, rather than being adopted from Caucasian norms in developed countries.
